# Crumbling Infrastructure and Learning Impairment: A Call for Responsibility

**DOI:** 10.1289/EHP69

**Published:** 2016-05-01

**Authors:** Edward D. Levin

**Affiliations:** Duke University, Durham, North Carolina, USA

We have known for many decades that lead exposure in children causes long-lasting cognitive dysfunction (Byers and Lord 1943; McKhann 1932). A great number of basic and human health studies have shown in great detail the neural mechanisms and behavioral consequences of developmental lead intoxication and how early-life lead exposure can lead to lifelong cognitive and emotional impairment. We have learned that even quite low levels of lead exposure can cause persistent cognitive impairment (Lanphear et al. 2005).

The removal of lead from gasoline and paint was one of the great successes of the environmental health movement (Needleman 1998), and lead levels in our children have declined steadily over recent decades (Jones et al. 2009). However, that improvement has stopped in recent years (NCEH 2016) and in some places reversed, like in Flint, Michigan, where a deteriorating public water system has resulted in elevated lead levels in people’s drinking water (Hanna-Attisha et al. 2016). Deferred maintenance and deteriorating infrastructure do not just result in the nuisance of leaking pipes and potholes, but also cause increased risks to human health. Lead neurotoxicity is not as immediately catastrophic as collapsing bridges, but is often more insidious, impairing cognitive and emotional function over a period of decades. Abdicating our social responsibility to maintain our nation’s infrastructure not only results in the obvious crumbling buildings and bridges, it also jeopardizes the brain development of our next generation.

As Dr. David Bellinger recently detailed in his excellent perspectives piece in the *New England Journal of Medicine* (Bellinger 2016), the excessive lead in Flint’s drinking water is “an abject failure to protect public health.” We know better and should be better stewards. We risk leaving our children not only a compromised environment but also compromised minds. The lead-laden drinking water in Flint is not only an environmental health crisis that must be remedied, it is also a call to be vigilant to prevent harmful exposures due to crumbling infrastructure, which can considerably add to the neurotoxic risks posed by other environmental toxicants found in and around our homes.

The cost of keeping our water (and air and food) clear of toxics pales in comparison to the cost of our impaired brain and health function over the decades to come. It is quite short-sighted to say that we cannot afford to maintain our infrastructure and environment, when in fact we cannot afford not to do so. Can we learn the lesson of Flint and come clean with our environment? Lead intoxication during development causes cognitive impairment. However, even those of us older folks who grew up in the era of higher lead levels should be able to learn the lesson of Flint and develop cleaner solutions for the drinking water of our children.
